# Extracellular Vesicles Physiological Role and the Particular Case of Disease-Spreading Mechanisms in Polyglutamine Diseases

**DOI:** 10.3390/ijms222212288

**Published:** 2021-11-13

**Authors:** Ricardo Moreira, Liliana S. Mendonça, Luís Pereira de Almeida

**Affiliations:** 1 CNC—Center for Neuroscience and Cell Biology, University of Coimbra, 3004-504 Coimbra, Portugal; ricardo.gomoreira@gmail.com; 2 CIBB—Center for Innovative Biomedicine and Biotechnology, University of Coimbra, 3004-504 Coimbra, Portugal; 3 Faculty of Pharmacy, University of Coimbra, 3000-548 Coimbra, Portugal

**Keywords:** extracellular vesicles, disease spreading, neurodegenerative diseases, polyglutamine diseases, vehicle, biomarker

## Abstract

Recent research demonstrated pathological spreading of the disease-causing proteins from one focal point across other brain regions for some neurodegenerative diseases, such as Parkinson’s and Alzheimer’s disease. Spreading mediated by extracellular vesicles is one of the proposed disease-spreading mechanisms. Extracellular vesicles are cell membrane-derived vesicles, used by cells for cell-to-cell communication and excretion of toxic components. Importantly, extracellular vesicles carrying pathological molecules, when internalized by “healthy” cells, may trigger pathological pathways and, consequently, promote disease spreading to neighboring cells. Polyglutamine diseases are a group of genetic neurodegenerative disorders characterized by the accumulation of mutant misfolded proteins carrying an expanded tract of glutamines, including Huntington’s and Machado–Joseph disease. The pathological spread of the misfolded proteins or the corresponding mutant mRNA has been explored. The understanding of the disease-spreading mechanism that plays a key role in the pathology progression of these diseases can result in the development of effective therapeutic approaches to stop disease progression, arresting the spread of the toxic components and disease aggravation. Therefore, the present review’s main focus is the disease-spreading mechanisms with emphasis on polyglutamine diseases and the putative role played by extracellular vesicles in this process.

## 1. Introduction

Extracellular vesicles (EVs) are a class of cell membrane-derived vesicles, which are believed to participate in several physiological and pathological pathways. EVs include a set of described cell-derived vesicles such as exosomes, microvesicles, ectosomes, apoptotic bodies, and other similar structures that have been described over the years [1,2,3].

In the mid 1960s, an interesting study referred to the term “platelet dust” shed by platelets [4], which can be considered the first description of microvesicles. Regarding exosomes, the first reports specifically mentioning them are from 1983, when two distinct studies described exosomes’ involvement in the recycling and elimination of transferrin receptors excess from reticulocyte cell membrane during their maturation [5,6]. Nevertheless, in the dawn of their discovery, exosomes were considered cellular waste with no major physiological functions. Therefore, the scientific community disregarded these particles for decades. Only in the late 1990s did two publications project EVs again in the spotlight, describing them, for the first time, as being crucial for intercellular communication, namely in B lymphocytes and Dendritic Cells (DC’s) [7,8]. In these studies, the presence of Major Histocompatibility Complex (MHC)-II proteins in EVs that specifically stimulated CD4^+^ T cells in vitro and further suppressed tumor growth in vivo was shown, demonstrating EVs as crucial structures in immune modulation.

Currently, it is well established that EVs are secreted by eukaryotic and prokaryotic cells, either as a communication or defense system [9,10], confirming that they are an evolutionarily conserved cellular mechanism. Moreover, analyzing the literature, EVs are described to be released by all cell types and can be found in biofluids, such as milk, saliva, blood, urine, and semen [1,11,12,13]. These particles carry proteins, lipids, and RNAs that might be used as biomarkers for diseases. Their physiological nature and the fact that their content is relatable to the state of the cell of origin [14] hints an interesting potential use for EVs as biomarkers of disease progression and therapeutic activity.

Brain imaging (e.g., MRI and PET) is currently considered the gold standard in disease progression evaluation. Considering that these methods are expensive and not ideal for early stage diagnosis of neurodegenerative diseases, CNS-derived EVs secreted into peripheral fluids appear as potential biomarkers to establish disease diagnosis long before the first symptoms [15] and to supply information regarding the disease progression during clinical trials.

Regarding Polyglutamine diseases, the association between the physiopathological mechanisms and EVs is not well established yet. However, the hypothesis that mutated proteins and their cleaved fragments or mutant mRNAs are spread across the brain via EVs is building evidence [16].

## 2. Pathological Spreading in Neurodegenerative Diseases

Neurodegenerative diseases have several clinical characteristics and neuropathological processes in common. Loss of autonomy, dementia, and movement impairments are clinical features exhibited by the vast majority of patients suffering from a neurodegenerative disease [17,18]. Neuropathologically, protein-specific aggregates, brain inflammation, and oxidative stress are neuropathological hallmarks present in several neurodegenerative diseases [17,19,20]. Protein inclusions, in particular, are relevant hallmarks of neurodegenerative diseases and are specific for each pathology; for example, amyloid-β (Aβ) and tau are hallmarks in Alzheimer’s disease (AD), α-synuclein in Parkinson’s disease (PD), mutant huntingtin (mHTT) in Huntington’s disease (HD), and mutant ataxin-3 in Spinocerebellar Ataxia type 3/Machado–Joseph disease (SCA3/MJD) [17,18,21]. In Amyotrophic Lateral Sclerosis (ALS), the misfolded Cu/Zn-superoxide dismutase (SOD1) protein was the first protein disfunction associated with familiar ALS. More recently, the transactive response (TAR) DNA binding protein 43 (TDP-43) was also implicated in the disease. Interestingly, TDP-43-positive ubiquitinated inclusions are present in almost all cases of ALS, both sporadic and familiar. However, it remains controversial whether TDP-43-positive inclusions are enrolled in the SOD1-related ALS pathology [22,23,24].

Of note, structurally, these aggregates have several common characteristics such as an abnormal enrichment in β-sheet structures, filamentous nature, and susceptibility to proteolytic cleavage, giving origin to oligomers even more prone to forming inclusions [25]. Moreover, in these types of diseases, there is typically one specific brain region or group of regions that is/are primarily affected, depending on the disease. Observations hint that, from these initial focal points, the neuropathology spreads across the brain in a topographically predictable manner, depending on the disease and its progression [26,27,28,29].

Polyglutamine diseases, such as HD and SCAs, are genetic neurodegenerative disorders characterized by an over repetition of the cytosine-adenine-guanine (CAG) trinucleotide which encodes for the glutamine amino acid (Q), resulting in an aberrantly extended polyQ tract in the disease-specific protein [30,31]. This overlong polyQ chain grants the protein a toxic gain of function and the propensity to aggregate [32]. Consequently, the mutated proteins impair several physiological pathways, such as cell waste clearance (autophagy and ubiquitin-proteosome system [UPS]), transcriptional functions, calcium homeostasis, and mitochondria functions [33,34,35,36,37]. Ultimately, these diseases are characterized by extensive neurodegeneration in multiple brain regions and patients’ symptoms are mostly related to neuromotor impairments. Moreover, the expansion of the polyQ tract and the age of disease onset and the disease severity are inversely correlated [38].

Analogously to the other neurodegenerative disorders, in Polyglutamine diseases, and particularly in HD, it has been investigated whether spreading of huntingtin would contribute to HD pathogenesis. In one of these studies it was demonstrated that several cell types are able to internalize synthetic polyQ aggregates, which invaded the nucleus or remained in the cytoplasm, promoting the formation of new aggregates [39]. Moreover, post mortem analysis of brain tissue from HD patients that were transplanted with fetal neural allografts found mHTT inclusions in the transplanted grafts one decade after the procedure [40].

**Figure 1:**
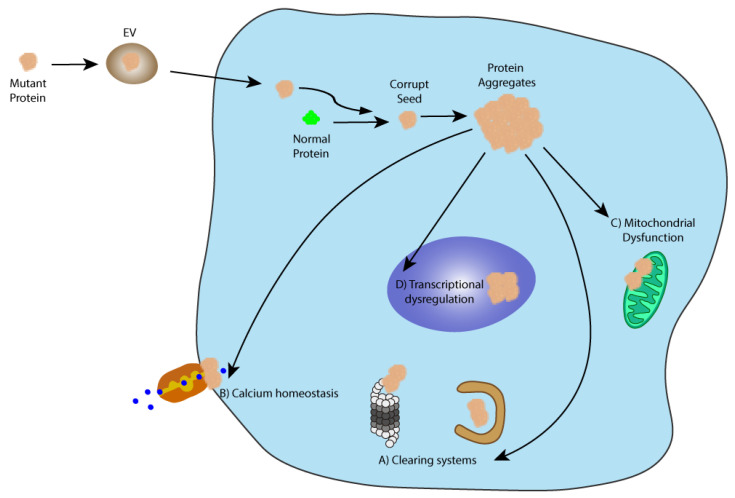
The prion-like seeding hypothesis. This theory states that like prions, misfolded disease-specific proteins may corrupt normal proteins and induce disease propagation via aggregation. One of the possible spreading mechanisms of these pathological seeds is via EVs. When in the cell, the corrupt seed may be able to induce misfolding in the endogenous proteins leading to the formation of protein aggregates that impair several key cellular pathways such as (A) clearing systems (autophagy and the UPS); (B) calcium homeostasis; (C) mitochondrial function, and (D) transcriptional dysregulation.

Evidence of a spreading mechanism in other polyglutamine disorders, particularly SCAs, are scarce, with one in vitro study suggesting that cells have the ability to internalize synthetic polyQ fragments. Once in the cells’ cytoplasm, these fragments induced proteins aggregation and interacted with components of the UPS, entrapping them in the aggregates. The inclusions endured several cell passages, indicating that the seeding system is self-sustained [39]. Lasagna-Reeves and collaborators demonstrated seeding and spreading of mutant ataxin-1 oligomers in a SCA1 mouse model. It was observed that when injected in the deep cerebellar nuclei of wild-type, transgenic null ataxin-1 mice or transgenic mice expressing mutant 78Q ataxin-1, cerebellar extracts from transgenic mice expressing mutant ataxin-1 with 154Q are able to induce a two to three-fold increase in ataxin-1 oligomers, but only in the transgenic mice expressing ataxin-1 with 78Q. These results indicate that mutant 154Q ataxin-1 is able to induce ataxin-1 oligomer formation in transgenic mice predisposed by expression of mutant polyQ ataxin-1. The authors also observed the propagation of these oligomers to neighboring cells but not to distal regions [41]. Additionally, a “Prion-like Domain” was identified in ataxin-1 and ataxin-2 proteins, which potentially make these proteins prone to induce seeding and aggregation [42]; nevertheless, no experimental evidence of a direct correlation between this domain and protein seeding and aggregation was provided. Despite these studies, the disease-seeding and spreading hypothesis (Figure 1) in SCAs need further studies.

### 2.1. Spreading Mechanisms

**Figure 2:**
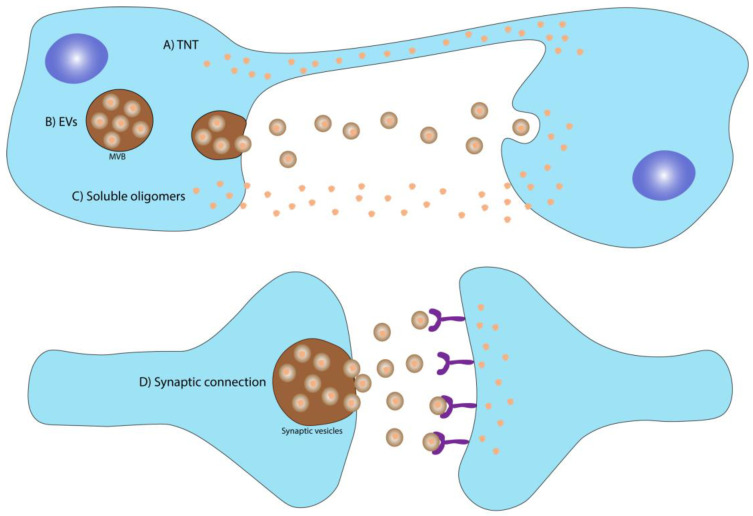
Spreading mechanisms of disease-associated agents in neurodegenerative diseases. The upper panel illustrates different mechanisms through which the disease-causing proteins/RNAs may spread from cell to cell: (A) tunneling Nanotubes, which are tunnel-like membrane-derived structures that connect two cells allowing the direct exchange of biomolecules; (B) extracellular vesicles that are membrane-derived vesicles secreted by cells with part of their cellular content, which can spread and deliver their content to distant cells; (C) soluble oligomers, which due to their small size may be able to escape the cell and spread to the extracellular milieu, and (D) synaptic connection, a highly effective communication pathway that may also be responsible for the transmission of disease-causing proteins between neurons.

For cytotoxic spread to occur, the disease-inducing agent needs to evade the cell of origin and reach the acceptor one. The presence of α-synuclein, Aβ, and tau in CSF and blood is evidence that these disease-causing factors are able to escape brain cells [43,44,45,46,47] to participate in transcellular dissemination of “prion-like” seeds. Several spreading mechanisms are considered to play a key role in the pathological transmission of these seeds, namely (i) soluble oligomers; (ii) synaptic connection; (iii) tunneling nanotubes, and (iv) extracellular vesicles (Figure 2) and there is no evidence to establish dominance of one spreading mechanism over others.

#### 2.1.1. Soluble Oligomers

The role of protein aggregates in neurodegenerative diseases is extensively reported and characterized. However, evidence that soluble oligomers of the disease-causing proteins cause more cellular toxicity and are the key toxic species responsible for disease progression has been provided [45,48,49,50]. This hypothesis was already tested in several neurodegenerative diseases including MJD, where a direct correlation between the presence of mutant ataxin-3 fragments and disease severity was established [51,52,53,54]. Soluble oligomers of the pathological proteins have the ability to evade the cell and disseminate to adjacent cells, triggering their pathological effects in the invaded cells [55]. This mechanism has also been observed for Prions disease and AD [55,56].

#### 2.1.2. Spreading through Synaptic Connection

Neurons rely on intercellular communication, which is achieved through electrical and chemical signals. Synapses are highly specialized junctions between presynaptic and postsynaptic cells, where after an action potential, neurotransmitters are released and can excite or inhibit the postsynaptic cell from firing its action potential [57,58]. Having this in mind, synaptic connections have been proposed as one possible route for transneural spread of disease-causing agents in the context of neurodegenerative diseases. Ahmed and colleagues observed a strong and fast propagation of aggregates upon infusion of hyperphosphorylated tau tangles into the hippocampus of human P301S tau transgenic mice. Interestingly, this spreading occurred preferentially to neurons that were synaptically connected, rather than to spatially close ones [59]. Likewise, in a donor–acceptor coculture method using either primary rat neurons or human differentiated SH-SY5Y cells (neuronal-like cells), spreading of oligomeric Aβ over the synaptic cleft was detected, leading to toxicity in the acceptor neurons that was dependent on the time of exposure [55]. Moreover, it was noticed that α-synuclein inclusions were taken up in nerve terminals, hinting that synaptic connection is key for the transfer of these inclusions. Once in the postsynaptic neuron, these aggregates induced impairment in synapse connectivity, leading to neuron death [60]. Regarding Polyglutamine diseases, Pecho-Vrieseling and colleagues showed that upon the transplantation of human embryonic stem cells (ESCs)-derived neurons into the brain of transgenic HD mice, mHTT spread from the host into the graft through synapses [61]. Babcock and Ganetzky using a transgenic *Drosophila* model of HD encoding mHTT with 138Q residues [62], observed an accumulation of mHTT aggregates in the synaptic terminals of the antennal lobe of the *Drosophila* central brain when mHTT was expressed in the olfactory receptor neurons. Over time, these aggregates spread to other brain regions resulting in neuropathology [63]. Thus, synaptic spread may accelerate the disease progression from more susceptible brain cells and regions to less disease-susceptible regions in PolyQ diseases [64], which might be a particularly important spreading mechanism in the case of ubiquitously expressed proteins, such as the mutant ataxin-3 in SCA3.

#### 2.1.3. Spreading through Tunneling Nanotubes (TNT)

Tunneling Nanotubes are transient tunnel-like membrane-derived structures, 50 to 700 nm in diameter and up to 100 μm long [65,66], that connect two cells, acting as a natural “highway” for the transfer of molecules between cells. These cellular structures are F-actin based and very versatile, responding to insults and stimuli. However, their fragile and short-lived nature, combined with the lack of specific molecular markers, make them difficult to study [67].

TNT have been shown to mediate prions transfer for its dissemination between neural cells [68], Aβ transfer from astrocytes to neurons when the cells were under stress [69], and dissemination of α-synuclein in SH-SY5Y cells and in primary pericytes isolated from post mortem PD patients [70].

Regarding polyQ diseases, in a study exploring the intercellular spreading of mHTT, a rapid transfer of polyQ aggregates between cells most likely mediated by TNT was observed in cocultures of CAD cells (between CAD cells expressing mHTT and control CAD cells) and in cocultures of murine cerebellar granule neurons transfected or not with mHTT fragments. The transmission required cellular contact and polyQ aggregates were found inside the TNT. Of note is the observation that mutant fragments, but not wild-type fragments, increased the number of TNT that further contributed to the pathological dissemination of the fragments to adjacent neurons [71].

#### 2.1.4. Spreading through Extracellular Vesicles (EVs)

Widely explored in recent decades, EVs have been linked to different physiological mechanisms. Several publications confirmed the presence of pathological proteins and RNAs in the EVs cargo, as well as the role of EVs as disease-spreading vehicles in neurodegenerative diseases [15,72,73,74,75,76] (Table 1).

Once in the EVs, these disease-related molecules can disseminate to distant cells, serving as seeds for pathological mechanisms activation in the acceptor cells and further contributing to disease spreading and progression. Considering EVs’ physical and physiological (small size, enrolled in cell communication, and made of self-cell membrane components) features, they are efficient vectors. In fact, they are endogenous nanoparticles able to evade the immune system, securing long circulation times and molecules transport to distant cells, while protecting their cargo from extracellular conditions, such as low pH and proteases [72,73,74,75,76].

EVs are highly versatile and dynamic cell derived lipid bilayer structures usually enriched in cholesterol, sphingomyelin, and ceramide, with the ability to serve as vehicle of several biomolecules, such as proteins, RNAs, and lipids [109]. Their dynamic nature allows EVs to be the result of stimuli or insults suffered by the cell of origin, and consequently, their cargo is highly influenced by the cell context [14]. It is noteworthy that, using advanced sequencing techniques such as next-generation sequencing, it was observed that the vast majority of RNA molecules present in EVs are small non-coding RNA (miRNA, siRNA, and iRNA), drawing attention to the regulatory gene expression function that EVs might have in target cells [110]. The huge amount of information available in this regard was compiled in online databases, such as ExoCarta (http://www.exocarta.org (accessed on 3 August 2021)) or Vesiclepedia (http://microvesicles.org (accessed on 3 August 2021)) [111].

Presently, there are no specific markers that precisely characterize and distinguish the different types of EVs. Usually, what is referred to is an enrichment of some markers, typical of each different type of vesicle [112]. Often, this specific enrichment is a consequence of the biogenesis process of the particles. Accordingly, these markers are frequently molecules of the ESCRT (Endosomal Sorting Complex Required for Transport) machinery, such as Alix, Flotilin-1, TSG-101, tetraspanins (CD9, CD63 and CD81), and lipid raft glycoprotein components, such as phosphatidylserine [1,72,113] (Table 2).

EVs have been explored as a potential source of biomarkers for diagnostics, and disease progression and response to therapy evaluation [15,72]. For example, the decreased levels of miRNAs (e.g., miR-193b) that specifically target APP mRNA, blocking its translation, in EVs from blood and cerebrospinal fluid (CSF) of AD patients, compared with those from patients in early stages of disease or control individuals, highlights the potential of EVs as biomarkers [114]. Additionally, it has also been demonstrated that EVs carry disease-related molecules between cells, causing neuropathological disease to spread [15,21,25]. On the other hand, there are studies demonstrating that EVs are enrolled in neuroprotective mechanisms. Accordingly, Yuyama and colleagues described that when EVs were injected into the brain of transgenic AD mice (with amyloid precursor protein [APP]) they acted as scavengers for toxic Aβ protein [115]. This hypothesis that EVs display neuroprotective functions was strengthened by the presence of Cystatin C (which is believed to trigger neuroprotection in the context of AD) in EVs derived from primary mouse neuron cultures [116].

## 3. Extracellular Vesicles Biogenesis

### 3.1. Exosomes Biogenesis

**Figure 3:**
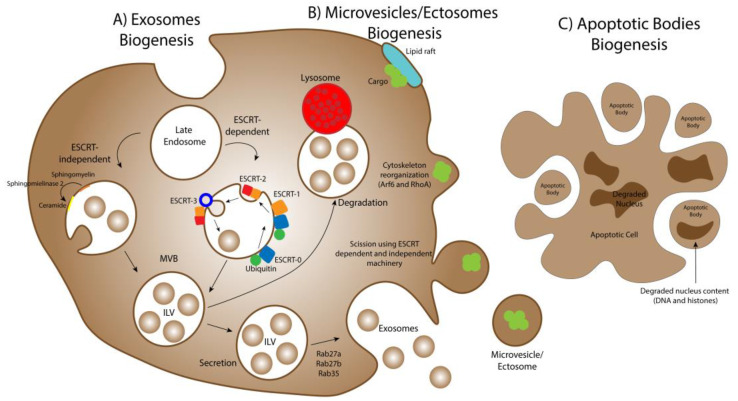
Biogenesis mechanisms of extracellular vesicles. (**A**) Exosomes biogenesis is governed by two main molecular mechanisms: the Endosomal Sorting Complex Required for Transport (ESCRT)-dependent and the ESCRT-independent pathways. In the first mechanism, several molecular rearrangements of the ESCRT machinery lead to the curving and scission of the membrane of the late endosome, creating the Intraluminal Vesicles (ILVs). In the second mechanism, the biochemical metabolization of sphingomyelin to ceramide by the enzyme sphingomyelinase 2 induces the formation of the ILV. After these processes, the Multivesicular bodies (MVB) either fuse with the lysosomes, degrading their luminal content, or fuse with the cell’s membrane, releasing the ILVs as exosomes in the extracellular milieu. (**B**) The biogenesis of microvesicles/ectosomes starts with the gathering of their cargo in the inner leaflet of the plasma membrane in specific regions’ designated lipid rafts (enriched in cholesterol and glycosphingolipids). Then, the cell’s cytoskeleton reorganizes, influenced by Arf6 and RhoA proteins, and the outward budding of the microvesicles starts. The ESCRT-dependent and -independent mechanisms described for exosomes biogenesis also play a role in inducing the curvature of the plasma membrane and scission, resulting in the pinching off of the microvesicles. (**C**) Apoptotic bodies biogenesis originates from the disassembly of cells undergoing apoptosis, through cytoskeleton rearrangements that cause their blebbing from the dying cell, entrapping organelles and pieces of the degraded nucleus, namely DNA and histones.

Exosomes have origin in late endosomes; using a complex network of molecular machinery, the cell membrane of those endosomes suffers inward budding, forming small structures called Intraluminal Vesicles (ILV) inside of a larger structure termed Multivesicular Bodies (MVB). Once matured, the MVB may either fuse with the plasma membrane, releasing ILV as exosomes in the extracellular medium, or with a lysosome, degrading its content [117]. The factors involved in the choice of the MVB for one of those fusions remains unclear. However, some evidence suggests that cholesterol content may be relevant in the MVB final destination [118]. Two distinct pathways can mediate MVB formation: the Endosomal Sorting Complex Required for Transport (ESCRT)-dependent pathway and the ESCRT-independent pathway (Figure 3).

The ESCRT-dependent pathway is a complex multimolecular network involving four subunits: ESCRT-0, ESCRT-I, ESCRT-II, and ESCRT-III. In this pathway, an initially ubiquitinated cargo is identified by ESCRT-0, -I, and -II, and invagination of the late endosome’s membrane is promoted by curvature-inducing factors that constitute ESCRT-I and ESCRT-II. Then, the binding of ESCRT-III to ESCRT-II prompts the deubiquitination of the tagged cargo and ubiquitin molecules release before cargo sequestration into MVB. Then, ESCRT-III drives vesicles budding, resulting in ILV production inside the lumen [119,120,121]. The ESCRT complex presents several proteins with ubiquitin-binding domains, which are key for the identification of ubiquitinated cargo and their integration in ILV within MVB biogenesis process [122,123,124]. Nevertheless, despite being the major sorting complex to MVB transport, the identification and sorting from the ESCRT machinery via ubiquitination is not the only mechanism for ILV cargo selection [124,125].

The ESCRT-independent pathway is the result of structural lipid modifications in the membrane of late endosomes. In particular regions of the membrane, a lipid raft occurs, with the metabolization of sphingomyelin to ceramide, by the enzyme sphingomyelinase 2, promoting the inward budding of the region and the formation of ILV [126]. Ultimately, in order to release ILVs to the extracellular medium, the MVB needs to dock and fuse with the plasma membrane. In this consideration, Rab GTPases, such as Rab27a, Rab27b, and Rab35 have been observed to be key players [127,128,129,130]. Once released by the cell to the extracellular medium, ILV are named exosomes [113]. Both ESCRT-dependent and -independent pathways seem to play a role in exosome biogenesis and the extent to which these pathways contribute to their biogenesis is highly dependent on the type and condition of the producer cell [3].

### 3.2. Microvesicles/Ectosomes Biogenesis

The biogenesis of microvesicles/ectosomes (Figure 3) is an erratic pathway that starts with the gathering of the EVs cargo in the inner leaflet of the plasma membrane in particular regions enriched in specific lipids such as cholesterol and glycosphingolipids, termed lipid rafts [109]. Afterwards, by spatial reorganization of the actin cytoskeleton prompted by Arf6 and RhoA, two proteins related with vesicular trafficking, cytoskeleton regulation, and plasma membrane rearrangement and recycling, an outward budding of the plasma membrane arises, resulting in the pinching off of microvesicles [131,132,133,134,135]. As previously observed, the hydrolyzation of sphingomyelin to ceramide by the enzyme sphingomyelinase 2, and molecular components of the ESCRT machinery are key factors in promoting the curvature and scission of the plasma membrane [1] (Table 2).

### 3.3. Apoptotic Bodies Biogenesis

Apoptotic bodies are the largest type of extracellular vesicles, with an estimated size between 1–5 μm in diameter [136]. These EVs present an identical biogenesis mechanism to microvesicles/ectosomes (Figure 3), given that both types of vesicles bleb from the plasma membrane as a consequence of molecular rearrangements in this structure [3]. Nevertheless, apoptotic bodies have origin in apoptotic cells, being the result of the apoptotic cell disassembly [137,138], which leads to the presence of intact organelles and complete pieces of DNA from the dying cell in these EVs [139]. Consequently, one characteristic marker of apoptotic bodies is the presence of histones in their lumen. Moreover, as a consequence of their irregular biogenesis process, apoptotic bodies usually are very heterogeneous in shape and size [140]. Moreover, as expected, their content highly depends on their cell of origin [141] (Table 2).

## 4. EVs Physiological Functions and Cargo Delivery to the Target Cells

**Figure 4:**
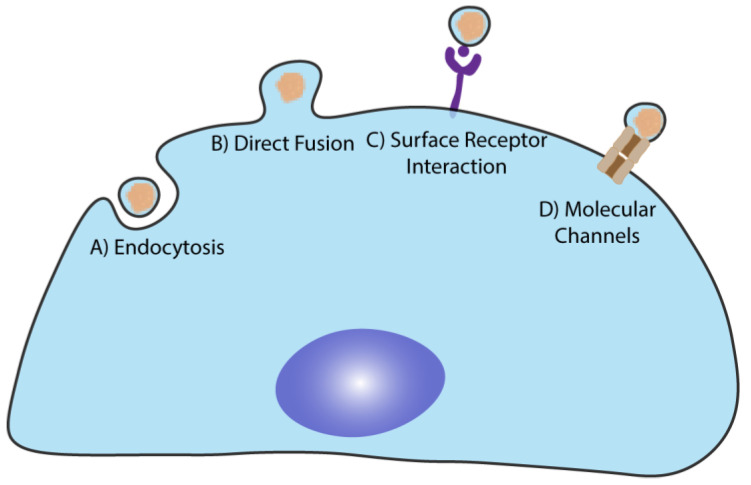
Cellular uptake pathways for extracellular vesicles (EVs). (A) EVs can be internalized by endocytosis, which is believed to be the main uptake pathway. EVs can also be internalized by (B) direct fusion with the plasma membrane of the target cell releasing their cargo into the lumen. (C) EVs can interact with the target cells without being internalized via surface receptor interaction and activation of signaling pathways. Finally, (D) the presence of molecular channels in EVs, such as connexin 43, may also promote the loading of the luminal cargo to target cells.

Several publications indicate that the target cell vs. EVs interaction is not random, occurring in a specific target—ligand mediated way (Figure 4) [142,143,144]. Numerous pathways and biomolecules have already been associated with the docking and cellular uptake of EVs. In some cases, the sole docking of the EVs to specific surface ligands present in the target cell is enough to induce a cellular response [144,145]. In other cases, it is essential that the EVs deliver their cargo to the intracellular environment [142,143,146]. Families of proteins such as tetraspanins, integrins, proteoglycans, connexins, and lectins orchestrate this protein interaction.

EVs are mainly internalized by endocytosis (clathrin-dependent and independent, pinocytosis, macropinocytosis, and calveolin-mediated internalization) and fusion with the target cell’s plasma membrane [143,147,148,149,150,151]. Moreover, in recent years it has been considered a possibility that molecular channels present in EVs can mediate the cargo transfer to target cells. An interesting study demonstrated that the Gap-junction protein Connexin 43 is expressed in EVs, forming functional channels and facilitating the transmission of the intraluminal content to the target cell [152]. Observations also suggest that EVs internalization occurs in a short period of time (as fast as 15 min after incubation), through an energy depending mechanism, as evidenced by the reduced internalization levels detected when the cells are kept at 4 °C, and is highly dependent on cytoskeleton dynamics [142,143,149,153,154].

Numerous cellular and molecular functions have been linked to EVs such as cell waste management, regulation of inflammation, cellular differentiation, coagulation, cell adhesion, and communication [140].

Immune modulation, as described above, was the first physiological function associated with EVs. In this regard, the presence of the death receptor agonist Fas ligand (FasL) in the surface of EVs allowed the interaction with T cells, activating apoptosis in these cells, consequently resulting in immune suppression. This EVs-mediated mechanism was described in cancer cells, where tumor cells release the EVs to evade the immune system and proliferate. It was also described in pregnancy, where the EVs prevent the mother’s immune system to recognize and attack the forming embryo [155]. Moreover, cancer cell-derived EVs are also able to suppress the immune system by inhibiting the proliferation of NK cells [156]. Chalmin and colleagues proved that the coadministration of dimethyl amiloride (a drug that inhibits EVs production) with the chemotherapeutic drug cyclophosphamide enhanced the efficacy of the anticancer drug [157], demonstrating the impact of EVs-mediated immune suppression in chemotherapy. Interestingly, depending on the context, EVs may as well present immune activation properties. For example, EVs isolated from synovial fibroblasts of rheumatoid arthritis patients exhibit tumor necrosis factor (TNF)-α in their surface, which delays T cell-mediated apoptosis [145].

Immune modulatory properties (stimulation or suppression) have also been associated with EVs derived from Antigen-Presenting Cells (APCs), such as Dendritic Cells (DCs), macrophages, and B cells [158]. Indeed, the fact that APC-derived EVs present on their surface the same proteins as their cells of origin, suggesting that they may have similar immune modulatory functions [159,160,161]. Commonly, EVs derived from APCs present factors such as the Major Histocompatibility Complex (MHC) I and II and adhesion molecules [7,8] promoting immune modulation when in contact with T cells, NK cells, macrophages, or other APCs [7,162,163].

The removal of cellular waste is one of EVs’ major functions and is important to maintain cell function, homeostasis, and ability to react to stress. For example, cancer cells are capable of loading anticancer drugs in their EVs, in this way disposing of them [164].

The role of EVs in inflammation has also been a topic of extensive research. EVs have been shown to transport key proinflammatory mediators such as Interleukin-1β (IL-1β) [165], tumor necrosis factor (TNF) alpha, and Interleukin-6 (IL-6) [166,167], and functional enzymes for the production of leukotrienes [168,169]. Moreover, the activation of inflammation through lipid components present in EVs was also demonstrated [170].

Additionally, EVs may also play important roles in the coagulation process [171], given that tissue factor (TF) and procoagulant phospholipids, mainly phosphatidylserine, are present in the membrane of EVs [172,173]. Furthermore, investigation of these two factors in EVs derived from blood of patients with multiple myeloma revealed that they are increased in the patients, as compared with control individuals, which might explain the increased risk of thromboembolism in cancer patients [174]. Tripisciano and coworkers observed an enrichment of TF and phosphatidylserine on the surface of platelet-derived EVs. These EVs, when added to EVs-free human plasma, were able to induce thrombin generation in a dose-dependent manner. Additionally, phosphatidylserine was demonstrated to increase coagulation by promoting elevated local concentration of coagulation factors [175].

Finally, neural regeneration and neurite outgrowth in neurons have been observed to be promoted by stem cells-derived EVs enriched in miR-133b [176]. This is a good example of the EVs’ capacity to promote cellular differentiation, particularly in the CNS.

Therefore, results indicate that depending on the physiological or pathological state of the origin cells, EVs have different cargo, and consequently activate different pathways when interacting with the target cell.

### 4.1. EVs in the Central Nervous System

Intercellular communication is fundamental for brain development, homeostasis, and adaptive response to external stimuli. In the past few years, the discovery that brain cells’ secret EVs [177] raised the hypothesis that these particles are key components in CNS development, function, and communication. Factors carried by EVs are known to play critical roles in neuronal plasticity, synaptic activity, and cell signaling [109]. For example, evidence indicates that Evenness Interrupted (Evi) protein (related to the Wnt family of morphogens enrolled in neurological processes such as brain development, cell migration and proliferation, differentiation, and plasticity [178]) can be secreted in EVs in the context of synapse formation and neuronal network development processes [179].

Antonucci and colleagues have shown that, at the synaptic level, the release of glutamate is influenced by microglia-derived EVs in a dose-dependent fashion. Additionally, the observed increase in synapse activity was independent of the EVs cargo, and rather promoted by the contact of the EVs via sphingolipid metabolism [180]. Furthermore, it was also observed that after glutamate stimulation, oligodendrocytes-derived EVs are released in a calcium-dependent mechanism, which are then internalized by neurons, protecting them from oxidative stress and starvation [181]. Indeed, neuron calcium equilibrium upon glutamate stimulation, mediated by NMDA receptors, seems to be a key aspect in EVs release by this type of cells. This fact is highlighted by experiments where calcium depletion, or inhibition of NMDA receptors, at the synaptic level, abruptly reduced EVs secretion [180,181].

The role of oligomannosidic glycans in brain development and function maintenance (via promotion of neurite outgrowth and synaptogenesis) is widely studied and well established. In this regard, Wang and colleagues demonstrated that the protein Synapsin I, a protein implicated in neural development and synaptic transmission due to its oligomannose-binding proprieties, is released via EVs by cultured cortical astrocytes under conditions of glucose deprivation or oxidative stress, hinting a neuroprotective role for these EVs [182].

Altogether, evidence demonstrates that EVs are carriers for different molecules enrolled in CNS development, synaptic function, and neuronal survival. Moreover, neural cell-derived EVs mediate active communication between brain cells and are able to specifically recognize other brain cells [109,183]. Nevertheless, EVs also play a role in pathological processes, such as neuroinflammation, given that the presence of inflammatory signals, such as IL-1β and COX2, in microglia-derived EVs may promote the reactivity of other microglia cells, increasing proinflammatory mediators’ production [184]. Additionally, as previously discussed, EVs have also been associated with the spread of molecules associated with neurodegenerative diseases [185,186].

#### The Particular Case of Polyglutamine Seeds Spreading

Regarding polyglutamine diseases, the information associating the spread of pathologic (polyQ) seeds and EVs is still very scarce. However, the first evidence that polyQ seeds may spread throughout the brain via EVs is emerging.

The presence of mHTT in EVs derived from human embryonic kidney (HEK) cells and from murine embryonic fibroblasts (MEF), which were exposed to stressful conditions (transfection with 84Q tract in HTT or proteasome blockage), was confirmed [187]. Authors observed that transglutaminase type 2 (TG2) interacts with components of the ESCRT machinery, such as Alix and TSG101, and is crucial for the recruitment of polyubiquitinated proteins such as mHTT into the EVs. Moreover, this recruitment was enhanced when the cell’s proteasome system was impaired, leading to the conclusion that EVs may be an alternative escape route to dispose of pathological proteins when the clearance mechanisms are compromised.

Wang and colleagues focused on the correlation between altered genes in HD and their EVs cargo content and observed an overlap of more than 50% between gene modifications and EVs cargo content [188]. In this work, the databases “HD Perturbation” and “HTT Interactome” (two databases that compile information regarding altered genes in HD and HTT protein network, respectively) were crossed; as expected, a high overlap between mHTT pathology and HTT biological functions was obtained. Furthermore, crossing the latter information with the “Exosome ProteinDB” database also resulted in a high overlap. These interesting results do not confirm, per se, the spread of mHTT via EVs but are strong evidence that EVs may play a crucial role in HD neuropathology.

Finally, Zhang and associates observed the presence of the polyQ tract of mHTT and its mRNA in EVs from HEK 293T cells expressing mutant HTT with different CAG repeat lengths. These EVs, carrying the polyQ tract of mHTT and its mRNA, were incubated with cultures of mouse striatal neurons resulting in the presence of the polyQ mRNA in their cytoplasm. Nevertheless, no evidence of toxicity was detected in the cells that internalized these EVs over the course of the experiment (72 and 96 h) [16].

## 5. Conclusions

In the past years it has been proposed that interneuronal seeding and spreading of disease-associated molecules occurs in several neurodegenerative diseases, analogous to the prion seeding mechanism present in Prions disease. It is hypothesized that pathological protein and/or RNAs evade the cell of origin and are captured by neighboring cells, inducing the pathology spread in the brain.

Several possible vehicles for this cell-to-cell spreading have been hypothesized. EVs —given their physiological proprieties, such as small size, versatile cargo capacity, ability to evade immune system and transporting molecules over long distances—appear as strong candidates. A handful of publications have already reported the presence of disease-causing proteins or RNAs of several neurodegenerative diseases, such as polyglutamine diseases, in EVs. Importantly, these EVs were able to induce the pathology both in vivo and in vitro experiments.

Consequently, the unequivocal demonstration of EVs involvement in neurodegenerative disease progression, and more insightful knowledge on the spreading mechanisms, might lead to the development and implementation of therapeutic strategies to block their release, or intake, by the target neurons to mitigate and treat neurodegenerative diseases progression.

## Figures and Tables

**Table 1:** Spreading mechanisms identified in neurodegenerative diseases.

**Disease**	**Spreading**	**Spreading via TNT**	**Spreading via EVs**
Creutzfeldt–Jakob disease	Guo and Lee, 2014 [25]; Thompson et al., 2016 [72]	Gousset et al., 2009 [68]	Fevrier et al., 2004 [77]; Vella et al., 2007 [78]; Cervenakova et al., 2016 [79]
Alzheimer’s disease	Baker et al., 1993 [80]; Clavaguera et al., 2009 [81]; Morales et al., 2012 [82]; Nath et al., 2012 [55]; Clavaguera et al., 2013 [83]; Clavaguera et al., 2014 [84]	Wang et al., 2011 [69]; Tardivel et al., 2016 [85]	Rajendran et al., 2006 [86]; Saman et al., 2012 [87]; Asai et al., 2015 [88]
Parkinson’s disease	Kordower et al., 2008 [89]; Desplats et al., 2009 [90]; Luk et al., 2009 [91]; Luk et al., 2012 [92]; Angot et al., 2012 [93]	Abounit et al., 2016 [94]; Dieriks et al., 2017 [70]	Emmanouilidou et al., 2010 [95]; Danzer et al., 2012 [96]; Stuendl et al., 2016 [97]
Amyotrophic Lateral Sclerosis	Mishra et al., 2020 [98]; Braak et al., 2013 [99]; Brettschneider et al., 2014 [100]; Smethurst et al., 2016 [101]; Pokrishevsky et al., 2016 [102]	Ding et al., 2015 [103]	Gomes et al., 2007 [104]; Grad et al., 2014 [105]; Basso et al., 2013 [106]; Silverman et al., 2019 [107]; Pinto et al., 2017 [108]
Huntington’s disease	Pecho-Vrieseling et al., 2014 [61]; Babcock et al., 2015 [63]	Costanzo et al., 2013 [71]	Zhang et al., 2016 [16]
Other PolyQ Diseases	Ren et al., 2009 [39]; Lasagna-Reeves et al., 2015 [41]	na	na

TNT = tunneling nanotubes; EVs = extracellular vesicles; na = not assessed.

**Table 2:** Physical and biological characteristics of exosomes, microvesicles/ectosomes, and apoptotic bodies.

	**Size (nm)**	**Biogenesis**	**Enriched Markers**	Sedimentation *	**Origin**
Exosomes	30–150	ESCRT-dependent or independent; lipid raft	Alix, CD63, CD81, TSG101 and flotilin-1	100,000 g	Late endosome MVB
Microvesicles/ Ectosomes	50–1000	Cytoskeleton reorganization and pinching off	CD40, PS	20,000 g	Plasma membrane
Apoptotic Bodies	1000–5000	Cytoskeleton reorganization and disassembly of apoptotic cells	Histones, DNA	10,000 g	Cells undergoing apoptosis

* centrifugation acceleration. g: gravitational acceleration; PS: phosphatidylserine; ESCRT: Endosomal Sorting Complex Required for Transport; MVB: Multivesicular Body.
